# Effects of Video-Game Based Therapy on Balance, Postural Control, Functionality, and Quality of Life of Patients with Subacute Stroke: A Randomized Controlled Trial

**DOI:** 10.1155/2020/5480315

**Published:** 2020-02-13

**Authors:** María José Cano-Mañas, Susana Collado-Vázquez, Javier Rodríguez Hernández, Antonio Jesús Muñoz Villena, Roberto Cano-de-la-Cuerda

**Affiliations:** ^1^Escuela Internacional de Doctorado, Universidad Rey Juan Carlos (URJC), Madrid, Spain; ^2^Hospital La Fuenfría (Servicio Madrileño de Salud), Cercedilla, Madrid, Spain; ^3^Department of Physical Therapy, Occupational Therapy, Rehabilitation and Physical Medicine, Faculty of Health Sciences, Universidad Rey Juan Carlos (URJC), Alcorcón, Madrid, Spain; ^4^Facultad de Formación del Profesorado, Universidad Autónoma de Madrid, Madrid, Spain

## Abstract

**Purpose:**

To determine the effects of a structured protocol using commercial video games on balance, postural control, functionality, quality of life, and level of motivation in patients with subacute stroke.

**Methods:**

A randomized controlled trial was conducted. A control group (*n* = 25) received eight weeks of conventional rehabilitation consisting of five weekly sessions based on an approach for task-oriented motor training. The experimental group (*n* = 25) received eight weeks of conventional rehabilitation consisting of five weekly sessions based on an approach for task-oriented motor training. The experimental group (

**Results:**

In the between-group comparison, statistically significant differences were observed in the Modified Rankin scores (*p* < 0.01), the Barthel Index (*p* < 0.01), the Barthel Index (*p* < 0.01), the Barthel Index (*p* < 0.01), the Barthel Index (*p* < 0.01), the Barthel Index (*p* < 0.01), the Barthel Index (*p* < 0.01), the Barthel Index (*p* < 0.01), the Barthel Index (*p* < 0.01), the Barthel Index (*p* < 0.01), the Barthel Index (*p* < 0.01), the Barthel Index (

**Conclusion:**

A protocol of semi-immersive video-game based therapy, combined with conventional therapy, may be effective for improving balance, functionality, quality of life, and motivation in patients with subacute stroke. This trial is registered with NCT03528395.

## 1. Introduction

Stroke constitutes a clinical syndrome with a rapid onset originated by a focal disorder of brain function of a vascular origin [1]. The global burden of stroke has continued to increase, representing an important public health problem and the second cause of death worldwide [2–4]. However, mortality after a stroke has decreased in recent years probably due to an improved control of risk factors, the recognition of stroke signs, an improvement in hospital care during the acute phase, and the development of strategies for secondary prevention, together with complementary interventions that offer comprehensive patient treatment [5].

Motor and sensitive deficits are common in stroke patients, producing disorders of motor control, balance, and gait [6]. In the subacute phase, alterations in body alignment occur, requiring the incorporation of treatment strategies focused on improving the postural control and symmetry of weight bearing [7–9]. In addition, limitations affecting the performance of activities of daily living are common, leading to an impact on patients' functionality and quality of life [10, 11].

Virtual reality (VR) and interactive video gaming have emerged as recent treatment approaches in stroke rehabilitation, with commercial gaming consoles, in particular, being rapidly adopted in clinical settings [4]. Key concepts related to VR are immersion and interaction. Immersion refers to the extent to which users perceive that they are in the virtual environment rather than the real world and is related to the design of the software and hardware. Virtual environments can vary in their degree of immersion of the user. Systems that include projection onto a concave surface or a head-mounted display are generally described as immersive, whereas a single screen projection is considered as semi-immersive and those using a desktop, joysticks, or pad displays are considered nonimmersive. Interaction with the environment can be made through a variety of simple devices, such as a mouse or joystick, or more complex systems using cameras, sensors, or haptic (touch) feedback devices. Thus, depending on the intervention, the user's level of physical activity may range from relatively inactive (for example, sitting at a computer using a joystick) to highly active (for example, challenging, full-body movements). Therefore, virtual reality relies on computer hardware and software to mediate the interaction between the user and the virtual environment.

The number of published studies involving the use of these technologies, such as video-game based therapy (VG), is on the rise, based on commercial game consoles, such as the Nintendo Wii®, the EyeToy by PlayStation®, or the Xbox with the Kinect® motion capture sensor for the treatment of stroke [12–14]. These systems facilitate the generation of movement, thanks to a certain level of immersion, as well as the interaction and simulation of human movement, via the performance of varied and progressive functional activities, with high levels of repetition and intensity, providing real-time multisensory feedback during task-oriented training, facilitating motor learning. In addition, VG produces improvements in the motor control of patients after a stroke with a positive impact on functional recovery [15–18]. The main basis for the use of video games is their ability to produce a plastic reorganization of the central nervous system, via the activation of adaptive neuroplasticity mechanisms, when virtual environments are used with appropriate levels of immersion that are both enjoyable and realistic [19, 20].

Furthermore, commercial video games adapted to neurological patients with functional deficits can promote motivation, self-esteem, and patient adherence to these interventions [12–14], through strategies that promote enjoyment, involving sensorimotor and cognitive specificity for the performance of the proposed tasks, and eliciting changes in motor control [21–24]. Nonetheless, few studies have been developed in subacute stroke patients with an appropriate methodological design, in order to develop clearly defined intervention protocols with commercial VG, as a complement to conventional treatment programs in terms of balance, postural control, functionality, quality of life, and motivation outcomes [25–27].

The aim of the present study was to examine the effects of a protocol based on commercial VG on balance, postural control, functionality, quality of life, and motivation outcomes in patients with subacute stroke. Our initial hypothesis is that a structured protocol, using the Xbox 360° video games console and the Kinect® device (video-game based therapy) designed by a neurorehabilitation team based on commercial VG, could be a complement to conventional therapy and correctly applied in a hospital environment for patients with subacute stroke.

## 2. Material and Methods

### 2.1. Design

A randomized controlled trial (RCT) was conducted. The participants were randomly distributed into two groups, using the QuickCalcs application by GraphPad Software®: a control group (*n* = 28) and an experimental group (*n* = 28). All participants had to be diagnosed with stroke in the subacute phase of illness, considered to be a period of between 15 days and six months after the vascular event [28].

Approval was obtained from the Ethics Committee of the ∗Blinded for peer review∗, conforming to the Helsinki Declaration. This trial was registered in ClinicalTrials with the register number NCT03528395.

All participants received a document informing them of the study aims and signed an informed consent. The directives of the CONSORT declaration for nonpharmacological RCTs were followed [29].

### 2.2. Subjects

In total, 56 subjects diagnosed with stroke in the subacute phase were initially recruited to take part in the study. All participants were patients hospitalized at the La Fuenfría Hospital (Madrid).

The inclusion criteria were patients of both sexes diagnosed with ischemic or hemorrhagic stroke confirmed by medical imaging, in the subacute phase, and aged between 18 and 80 years, with a score on the National Institute of Health Stroke Scale (NIHSS) [30] below 20, a Montreal Cognitive Assessment (MoCA) [31, 32] score equal to or above 14 (mild cognitive decline or absence of cognitive decline), a modified Rankin scale [33] score between 0 and 4, subjects able to maintain a standing position unassisted, and a score of ≥1 on the Functional Ambulation Categories (FAC) [34].

The exclusion criteria were determined by the presence of other visual, auditory, musculoskeletal, bone, or joint alterations in the acute or chronic phase that could influence the primary pathology; the presence of other neurological or cardiovascular illnesses which contraindicated physical exercise; patients unable to maintain a sitting position unassisted; subjects who, at any time, displayed a worsening state of health due to another medical problem; subjects who displayed a contraindication for the use of VG devices and commercial video games, such as the presence of photosensitive epilepsy, or a score above two in the extremities on the modified Ashworth scale [35]; and patients who were unable to collaborate, with behavioral disorders, or rejecting treatment with video-game based systems.

### 2.3. Assessments

Two assessments were performed. First, a pretreatment assessment was performed after assigning the subjects to the control or experimental group. Second, a posttreatment assessment was performed eight weeks after the intervention. All the assessments were performed with two evaluators who were blinded to the established study groups. The same environmental conditions were maintained during both assessments to limit the influence of external factors for both the assessments, and for the intervention. Both evaluators received previous training for the administration of the scales and tests used in order to guarantee the reliability criteria.

#### 2.3.1. Outcome Measures

All study participants were evaluated using the following outcome measures:

*Modified Rankin Scale*. This is a useful tool, which has been validated and translated to categorize the functional level after a stroke. This scale determines the level of physical disability based on a score from 1 to 5 [33]. This outcome measure was considered as the main outcome measure for the calculation of statistical power and to describe changes in the level of functional independence in the pre-post assessments of both groups.

*Barthel Index*. This test evaluates basic activities of daily living, validated in the context of stroke. In this study, we used the version translated and adapted to Spanish. The Barthel Index comprises 10 items: feeding, personal toileting, bathing, dressing and undressing, getting on and off a toilet, controlling bladder, controlling bowel, moving from wheelchair to bed and returning, walking on level surface, and ascending and descending stairs. The total score ranges between 0 and 100 (the lower the score, the greater the dependence) [36].

*Tinetti Scale for Balance and Gait*. This scale is validated for the assessment of balance and gait in the context of stroke, and has been translated and adapted to Spanish. The maximum score for balance is 16 and for gait 12, out of a total of 28 points. A greater overall score indicates a lower risk for falls (less than 19 equals a high risk of falls; from 19 to 24 is a moderate risk) [37].

*Functional Reach Test*. This test evaluates the dynamic balance. It is performed in the following start position: the patient in standing, placing the shoulder in 90° flexion with a closed fist. The healthy side is placed close to but not touching the wall, and the maximum anterior distance is measured without providing assistance. The patient is asked to lean forward, as far as possible, without moving the feet, and the end point is measured based on the position of the third metacarpal joint. The scores are determined via the assessment of the difference between the beginning and end positions. The reaching distance is noted in centimeters, and three tests are performed with a 15-second rest period between each. The mean of the two last measures is taken. The established cut-off rate of individuals with stroke to determine the risk of falls is 15 cm of anterior reach [38].

*Get Up and Go Test*. This test assesses functional mobility and balance. For this study, we used a version adapted and translated into Spanish. The person is asked to stand up from a chair without using the arms, walk three meters forward in a straight line, turn around, return, and sit down again. The test is scored from 1 to 5, with 1 being normal and 5 almost falling during the test, administered by supervising the patient on one side [39].

*Baropodometry*. A static test in standing was performed using the T-plate ® pedometer (T-plate foot pressure plate model, Medicapteurs, BA, France), which provides information on the pressure exercised by each point on the sole of the foot. The distribution of loads was registered using a force plate (%) and by calculating the support surface (cm^2^) of each foot, informing of the position of the center of pressure when the subject maintains unassisted standing for 10 seconds, gazing forward at a fixed point [40].

*EuroQoL 5D (EQ-5D)*. This is a self-administered, generic questionnaire, adapted to Spanish, which evaluates the health-related quality of life in five dimensions (mobility, personal care, activities, pain/discomfort, and anxiety/depression) to determine quality of life. This includes a visual analog scale (VAS), which is considered optimal to assess the perceived health status at the time of the test (0–100) [41].

*Scale of Satisfaction, Adherence, and Motivation with the Treatment of Video-Game Based Therapy*. This purposely designed questionnaire was designed by the research team as no validated and translated questionnaire existed to measure motivation in these types of video-game based interventions. This is a Likert scale administered to an experimental group before and after completing the protocol of commercial video games. The interpretation of the scale is as follows: the higher the scores, the higher the satisfaction (five items, score 25), self-esteem (five items, score 25), and adherence (six items, score 30). The total score ranges from 0 to 80 (Table 1).

The adverse effects of the treatment were recorded by interview at the end of each session together with the percentage of adhesion of participants in the experimental group receiving the video-game based therapy.

### 2.4. Intervention

The intervention protocol was applied by four therapists. Therapists were blinded to the participants' initial and final assessments.

#### 2.4.1. Control Group

The control group received eight weeks of conventional rehabilitation consisting of five weekly sessions comprising 45 minutes of physical therapy and 45 minutes of occupational therapy. It total, 40 sessions of physical therapy and 40 sessions of occupational therapy were administered, both of which were based on an approach for task-oriented motor training [42]. During these interventions, strategies were used to promote the development of activities of daily living (ADL) based on repetition, feedback, intensity progression, variation of interventions, and tools for the acquisition of motor requirements (trunk control in sitting, transfer from sitting to standing, standing with assistance and autonomy, weight transfers, single leg support, and reeducation of gait) [43, 44].

#### 2.4.2. Experimental Group

The experimental group received conventional rehabilitation (35 minutes of physical therapy and 35 minutes of occupational therapy) plus the experimental intervention (20 minutes), consisting of video-game based therapy during eight weeks with commercial video games using the Xbox 360° video games console and the Kinect® (Microsoft Corporation, Redmond, WA, USA) device, receiving three sessions per week on alternate days over an eight-week period, for a total of 24 sessions per participant. For the remaining days of the week without experimental treatment, the patients followed the conventional treatment scheduled. All experimental treatments were performed immediately after the conventional rehabilitation sessions. The intensity and motor requirements of each Kinect® session gradually increased. Thus, the Kinect® session progressed based on the sensorimotor requirements of the participant, and working in different positions, such as sitting, sitting combined with standing, and standing with and without help by physical therapists. The total treatment times for both groups were always the same throughout the entire intervention process (90 minutes per day for both groups).

**Figure 1:**
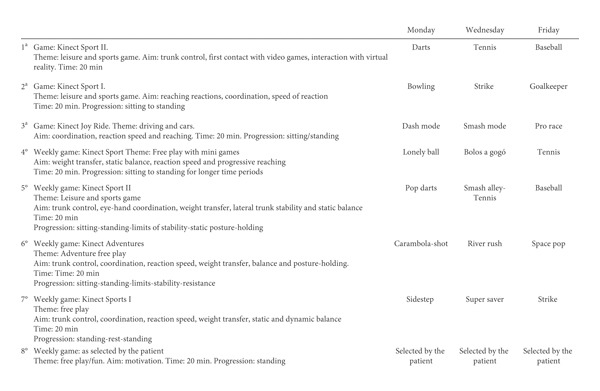
Video-game based protocol with Xbox® and Kinect®.

The therapeutic tool used was the Xbox 360° video-game console and the Kinect® device, using the following games: Kinect Sports I®, Kinect Sport II®, Kinect Joy Ride®, and Kinect Adventures®, based on a specific protocol (Figure 1), designed by three physical therapists with over 10 years of experience and one physiatrist with over 15 years of experience in the field of neurological rehabilitation with people with stroke and tested for patients with stroke in a previous pilot study. During the initial weeks, the protocol was focused on the patient's trunk control, reaching reactions, speed of reaction, and interaction with the upper limbs using the VG. The weekly progression was directed at facilitating autonomous standing with weight transfer work, limits of stability, upper limb control, and dynamic balance.

#### 2.4.3. Sample Size Calculation

The main outcome measure used to calculate the sample size was the modified Rankin scale [45]. The G∗Power 3.1.6 program was used for statistical analysis [46], considering that the estimated effect size for the main measure was 0.25. Considering a statistical power test of 0.95, an alpha error of 0.05, and a total of two measurements performed for the two groups, the estimated sample size required was 48 participants.

### 2.5. Statistical Analysis

The coding and treatment of data were conducted using the SPSS 22.0 statistical program for Windows. A descriptive analysis was performed (mean and standard deviation), considering the normal distribution (Kolmogorov–Smirnov) and measures of contrast (tests for differences in means). Regarding the comparison of intragroup means (pre- and posttreatment), the Wilcoxon test for paired data was applied to all variables that did not follow a normal distribution. In the case of variables that presented a normal distribution, Student's *t*-test was used. The difference of the means between groups in the variables without a normal distribution was calculated using the *U* Mann–Whitney test for independent samples, whereas Student's *t*-test was used for those with a normal distribution (between-group difference in means). The level of statistical significance was set at a *p* value of ≤0.05.

## 3. Results

**Figure 2:**
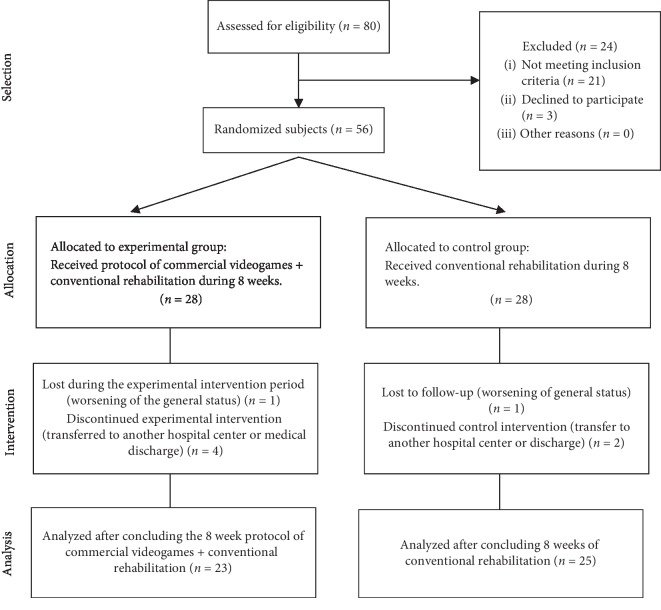
Flow diagram.

Initially, 80 prospective participants were identified. Of these, 56 participants fulfilled the inclusion criteria and were distributed between both groups. Ultimately, 48 patients finished the complete intervention. There were three dropouts in the control group (*n* = 25) and five dropouts in the experimental group (*n* = 23) (Figure 2). This was due to a worsening of their general health status and was not related to the type of intervention performed and/or transfers to another hospital center. No adverse event was registered derived from the treatment in any of the study groups.

The mean age ±standard deviation of the sample, comprising 25 women and 23 men, was 63.13 ± 10.38 years, aged 65.68 ± 10.39 years in the control group (14 women and 11 men), and 60.35 ± 9.84 years in the experimental group (11 women and 12 men).

The results related to the demographic variables of the sample are presented in Table 2. The variables of age, time of evolution post-stroke, NIHSS, and MoCA test followed a normal distribution. Statistically significant differences were observed between both groups for the variables on the affected side (*p*=0.03) and the MoCA test (*p*=0.01). The percentage of participants diagnosed with ischemic stroke was 60% in the control group and 73.9% in the experimental group. Regarding the affected side of the body, the left side was affected in 60% of participants of the control group and 87% of the experimental group. Concerning the previous management of technological tools, 68% of participants in the control group were familiar with the use of technology, compared to 69.6% in the experimental group. No statistically significant differences were observed for the remaining variables administered prior to the intervention period, with the exception of pain/discomfort, anxiety/depression, and VAS for perceived health status.

The results of the comparisons for the intragroup and intergroup variables are shown in Table 3. Regarding the intragroup changes, significant improvements were found in the control group for the Barthel Index variables (*p* < 0.01), the Tinetti gait (*p*=0.01) and balance test (*p* < 0.01), the Functional Reach test (*p*=0.03), the Get Up and Go test (*p*=0.03), and the anxiety/depression dimension (*p*=0.03) of the EQ-5D. In the experimental group, significant differences were found in the modified Rankin scores (*p* < 0.01), baropodometry (*p* < 0.01), and the variable related to strength and the pain/discomfort dimension (*p* < 0.01) of the EQ-5D.

For the intergroup variables, statistically significant differences were observed for the modified Rankin variables (*p* < 0.01), the Barthel Index (*p*=0.05), the Tinetti gait test (*p*=0.02), the Functional Reach test (*p* < 0.01), the Get Up and Go test (*p*=0.05), the pain/discomfort dimension (*p* < 0.01), and the anxiety/depression dimension (*p* < 0.01) of the EQ-5D and the VAS (*p* < 0.01) for the perceived health status according to the EQ-5D questionnaire.

The results obtained in the experimental group for the motivation, self-esteem, and adherence scale before and after receiving the video-game based protocol are shown in Table 4. Statistically significant differences were obtained for motivation (*p* < 0.01), self-esteem (*p* < 0.01), and adherence (*p* < 0.01). The percentage of assistance provided to participants from the experimental group was 95.28%, performing 526 interventions in a total of the 552 planned, during the eight-week duration of the experimental intervention.

## 4. Discussion

This RCT examines the effects of a structured protocol based on commercial video games combined with conventional rehabilitation for subacute stroke inpatients. Objective and validated outcome measures adapted to patients with stroke were used for the assessment of balance, postural control, functionality, quality of life, level of motivation, adherence, and satisfaction, compared with a control group. Our results show that the use of a structured protocol combined with conventional therapy produces significant changes at the level of physical disability, basic ADL, balance and gait capacities, functional mobility risk of falls, and health-related quality of life, producing significant changes on levels of motivation, self-esteem, and treatment adherence in patients who are hospitalized after a stroke (subacute phase).

Authors such as Ho et al. [46] suggest that the combination of rehabilitation based on VR and conventional therapy could be more effective for the acquisition of functional improvements in patients after stroke. These results are supported by systematic reviews [4, 44, 47] which indicate that VR produces a beneficial effect on ADL when it is used together with conventional approaches, such as in the present study. Along these lines, Gibbons et al. [48] indicate that VR interventions are, at least, as effective as conventional physiotherapy for improving the functional results of the lower limbs after a stroke, on the condition that the protocols that are used are made progressively more intensive considering both time and difficulty. Our results support this claim, showing intragroup benefits for the control and experimental groups, whereas only for the experimental group in intergroup comparisons in terms of balance, postural control, functionality, quality of life, level of motivation, adherence, and satisfaction. Therefore, intensity, a progressive difficulty of the tasks in VR environments, and the combination of conventional and VG approaches should be considered for the rehabilitation of individuals with subacute stroke.

Several systematic reviews have been published on the use of VR, via the use of game consoles, which inform of significant changes in the motor function of patients after a stroke, with the use of specific implemented protocols [12], as used in our research. However, prior research indicates that protocols with low times and intensity, as well as a low number of sessions and weeks of treatment, do not produce significant differences in outcome measures (<30 min/session, <3 sessions/week, with a frequency <4 weeks and unsupervised treatment) [49, 50]. This is why our protocol was structured based on three sessions per week over an eight-week period (24 sessions per participant) and always supervised by physical therapists.

Our results reflect improvements in the performance of ADL and physical disability. Lee et al. [51] employed a similar protocol to that used in the present study, via the Kinect® system, with a lower number of sessions and weeks, however with the same commercial video games used in the current work. They did not find improvements in the ADL assessed by the Modified Barthel Index of stroke patients. A possible explanation for these results is that the dose and the possibility of reaching a high intensity of repetitive and specific practice providing a multisensory feedback are important to obtain ADL modifications in subacute stroke patients, as we show in our study. Furthermore, other works point to the need to indicate the risks of the use of these devices, such as cyber sickness, pain, or falls. Recommendations show that stroke survivors are able to tolerate 30–60 mins 3 to 5 times per week (an average of 180 mins gaming per week) without experiencing significant adverse effects [52]. In our study, our results are in line with these recommendations as no adverse effects were found to be derived from the experimental intervention. Thus, the use of video-game therapy based commercial gaming can provide high-intensity practice without risk for patients as long as they are supervised and have some previous familiarization with such technology.

We found improvements in the Tinetti gait test, the Get Up and Go test, and the Functional Reach test, indicating that the use of commercial video games using the Kinect® device, combined with conventional therapy, can be considered an effective tool for improving balance and postural control, with a potential effect on the decreased risk of falls in patients who have suffered a stroke. After consulting the scientific literature, we were unable to find similar protocols, based on the use of VG for the study of the effect of these on the risk of falls in patients with stroke. There are several possible explanations for these improvements in dynamic balance, such as the possibility of working on active trunk control, as well as facilitation of reaching reactions and the speed of reaction, all of which are aspects related to the acquisition of appropriate postural control [6–9]. Surprisingly, no improvements were found for the Tinetti balance test. This could be because both treatment modalities are effective for balance recovery in subacute stroke patients; however, the semi-immersive video-game approach could present more benefits for dynamic balance assessed by the Tinetti gait test, Functional Reach test, and Get Up and Go test. Some studies affirm that when VR is combined with conventional therapy, it is moderately more effective at improving dynamic balance than conventional therapy alone in patients after a stroke [51–56]. A recent systematic review has suggested that VR interventions using protocols based on more than 10 sessions may have a positive impact on dynamic balance and the recovery of gait [57]. Therefore, our results could be justified again by the dose used and the type of activities retrained.

Body alignment and symmetry were assessed using a baropodemetry system to determine changes in postural control. No significant results were found for this outcome in both groups, and these findings may be due to the variability between patients. However, the pre- and postintervention results in the experimental group revealed statistical significance for body alignment and foot symmetry, in relation to the distribution of loads on the force plate. Future studies should establish the effect of video-game based therapy on postural control in patients with subacute stroke, measured using this quantitative postural control tool.

Concerning quality of life, our results indicate that the combination of conventional treatment with a semi-immersive video-game approach produced positive effects on the perception of pain/discomfort, sensation of anxiety/depression, and an increased subjective perception of patients regarding their health status. The use of video games may be an appropriate complement to the conventional rehabilitation of subacute stroke; however, it is necessary to develop protocols that consider the stages of motor learning, involving high practice intensity, positive feedback between stimulation response and increased motivation [4]. A recent meta-analysis correlated depression after a stroke with a significantly greater risk of mortality in patients post-stroke; therefore, it is essential to establish lines of research for decreasing anxiety and pain in these patients. Along these lines, the use of video games may be an interesting tool for inclusion in adapted protocols for patients with stroke [58].

Our findings for motivation, adherence, and satisfaction, assessed in the experimental group, revealed significant results after the protocol of semi-immersive video games. The scale used presents the limitation of the lack of validation; however, to our knowledge, no similar tool has quantified these dimensions related to the use of technology in the neurological patient. Other authors [47, 59] suggest the need to study the potential acceptability and effectiveness of commercial video games to obtain motivation-related outcomes. In addition, for some authors, the principal characteristics of these interventions are the low cost of the system, its portability, and high levels of acceptance on behalf of patients. This research promotes the integration of these systems in clinical practice, hypothesizing that these systems may be viable for being incorporated to conventional treatments in patients with stroke as coadjuvant treatment [12, 50, 57, 60].

### 4.1. Study Limitations

Our study presents several methodological limitations, such as the lack of a long-term follow-up. This was not possible because the study was conducted at a hospital that was part of the public health system, and the participants were eventually discharged from the hospital. In addition, there was heterogeneity in the type of stroke included in the current work. It is important to highlight that a subjective scale was used to quantify motivation, adherence, and satisfaction. To our knowledge, no validated tool has been described for this purpose. Furthermore, the software employed was not specific for the management of patients with stroke. Finally, the results of the present work cannot be extrapolated to other patients with stroke in other stages of the illness, and future research should validate this protocol alongside other rehabilitation strategies.

## 5. Conclusions

Our results suggest that the use of a video-game based protocol using commercial video games, combined with conventional therapy, may produce improvements of balance, postural control, functionality, quality of life, level of motivation, adherence, and satisfaction in patients with subacute stroke.

## Figures and Tables

**Table 1:** Scale of satisfaction, self-esteem, and adherence.

	0. No comments	1. Strongly disagree	2. Disagree	3. Uncertain	4. Agree	5. Strongly agree
1. I find the setup provided by a cutting-edge game console to be enjoyable and appealing						

2. These virtual environments awaken my interest as a complement to my conventional therapy						

3. The Xbox 360 Kinect® allows me to direct the activity by continuously interacting and receiving information						

4. This protocol is useful for improving functional capacities, balance, and postural control						

5. The virtual environment allows me to interact with interesting scenes						

6. I am able to do things well, like other people, adapting to my functional limitations						

7. There are times when I feel I am not useful, and that I cannot do the tasks appropriately						

8. At times I feel that I am unable to do what I am asked to do and I feel discouraged						

9. I am convinced that I have good qualities for improving my limitations						

10. These complementary interventions make me feel stressed and tired						

11. I feel that the number of virtual reality sessions that I receive per week is sufficient						

12. The recommendations/requests made by the therapist seem easy						

13. I consider that the time employed in this approach using game consoles is sufficient						

14. The therapist modulates the intensity at all times, according to my general status						

15. I would like to continue doing this type of activity because it motivates and interests me						

16. I have attended all sessions with eagerness and enthusiasm						

**Table 2:** Sociodemographic characteristics of the sample.

Variable (*N* = 48)	K-S^†^	Control (*n* = 25), mean ± SD	Experimental (*n* = 23), mean ± SD	*p*
Age (years)		65.68 ± 10.39	60.35 ± 9.84	0.11
Gender (male/female)		11/14	12/11	0.58
Type of stroke (hemorrhagic/ischemic)		10/15	6/17	0.31
Side of the body affected (right/left)		10/15	3/20	**0.03** ^*∗*^
Time of evolution of the stroke (days)	0.20	54.52 ± 18.74	50.91 ± 18.44	0.50
NIHSS^‡^	0.20	14.28 ± 4.13	13.17 ± 3.47	0.32
MOCA test	0.16	18.12 ± 3.74	22.26 ± 4.11	**0.01** ^*∗∗*^
Prior use of Xbox + Kinect technology (yes/no)		17/8	16/7	0.90

^†^K-S: Kolmogorov–Smirnov. ^‡^NIHSS: National Institute of Health Stroke Scale. Note: ^*∗∗*^*p* < 0.01,  ^*∗*^*p* < 0.05.

**Table 3:** Results of the comparisons of the intragroup and intergroup variables.

	K-S	Control (*n* = 25), mean ± SD		CI 95%, *p* value, intragroup	Experimental (*n* = 23), mean ± SD		CI 95%, *p* value, intragroup	CI 95%, *p* value, intergroup	
		Pre	Post		Pre	Post		Pre	Post
Modified Rankin (1–5)		3.92 ± 0.27	3.72 ± 0.68	*Z* = −1.89 0.05 to 0.06; **p**=0.05^*∗*^	3.91 ± 0.28	3.22 ± 0.60	*Z* = −3.77 0.00 to 0.00; **p** < 0.01^*∗∗*^	*Z* = −0.08 0.65 to 0.67; *p* < 0.93	*Z* = −3.25 0.00 to 0.00; **p** < 0.01^*∗∗*^

Barthel Index (0–100)	0.20	45.60 ± 19.96	56.60 ± 18.29	*t* = −7.20 −14.15 to −7.84; **p** < 0.01^*∗∗*^	45 ± 22.96	65.87 ± 16.21	*t* = −5.40 −28.87 to −12.86; **p** < 0.01^*∗∗*^	*Z* = −0.21 0.40 to 0.42; *p*=0.82	*Z* = −1.89 0.02 to 0.03; **p**=0.05^*∗*^

Tinetti gait (0–12)		2.40 ± 3.52	3.42 ± 4.26	*Z* = −0.27 0.00 to 0.00; **p**=0.01^*∗∗*^	3.04 ± 3.50	5.57 ± 3.14	*Z* = −3.17 0.00 to 0.00; **p** < 0.01^*∗∗*^	*Z* = −0.95 0.16 to 0.17; *p*=0.34	*Z* = −2.18 0.01 to 0.01; **p** < 0.02^*∗*^

Tinetti balance (0–16)	0.20	7.68 ± 4.32	9.84 ± 3.65	*t* = −6.26 −2.87 to −1.44; **p** < 0.01^*∗∗*^	8.61 ± 4.16	11.61 ± 2.85	*t* = −5.11 −4.21 to −1.78; **p** < 0.01^*∗∗*^	*Z* = −1.65 0.21 to 0.22; *p*=0.09	*Z* = −1.76 0.03 to 0.04; *p*=0.07

Functional Reach test		3.36 ± 5.59	4.20 ± 5.50	*Z* = −2.13 0.01 to 0.01; **p**=0.03^*∗*^	4.96 ± 5.74	11.04 ± 7.35	*Z* = −3.91 0.00 to 0.00; **p** < 0.01^*∗∗*^	*Z* = −0.77 0.04 to 0.05; *p*=0.43	*Z* = −3.60 0.00 to 0.00; **p** < 0.01^*∗∗*^

Get Up and Go test (1–5)		4.16 ± 0.85	3.88 ± 0.97	*Z* = −2.11 0.02 to 0.3; **p**=0.03^*∗*^	3.96 ± 0.97	3.43 ± 0.89	*Z* = −2.34 0.01 to 0.01; **p**=0.01^*∗∗*^	*Z* = −0.59 0.27 to 0.28; *p* < 0.55	*Z* = −1.88 0.03 to 0.03; **p**=0.05^*∗*^

*EuroQol-5D*									
Mobility (1–3)		2.72 ± 0.45	2.64 ± 0.56	*Z* = −1.41 0.23 to 0.25; *p*=0.15	2.61 ± 0.49	2.48 ± 0.51	*Z* = −1.24 0.17 to 0.19; *p*=0.21	*Z* = −0.80 0.29 to 0.31; *p*=0.41	*Z* = −1.24 0.12 to 0.13; *p*=0.21

Personal care (1–3)		2.72 ± 0.45	2.64 ± 0.56	*Z* = −1.41 0.23 to 0.25; *p*=0.15	2.61 ± 0.49	2.43 ± 0.50	*Z* = −1.52 0.09 to 10; *p*=0.12	*Z* = −0.80 0.29 to 0.31; *p*=0.41	*Z* = −1.52 0.07 to 0.08; *p*=0.12

Activities (1–3)		2.68 ± 0.47	2.60 ± 0.57	*Z* = −1.41 0.23 to 0.25; *p*=0.15	2.61 ± 0.49	2.43 ± 0.50	*Z* = −1.24 0.09 to 0.10; *p*=0.21	*Z* = −0.51 0.40 to 0.42; *p*=0.61	*Z* = −1.24 0.12 to 0.13; *p*=0.21

Pain/discomfort (1–3)		2.44 ± 0.71	2.20 ± 0.76	*Z* = −1.89 0.05 to 0.05; **p**=0.05^*∗*^	1.96 ± 0.76	1.48 ± 0.59	*Z* = −3.20 0.00 to 0.00; **p** < 0.01^*∗∗*^	*Z* = −2.19 0.01 to 0.02; **p** < 0.02^*∗*^	*Z* = −3.20 0.00 to 0.00; **p** < 0.01^*∗∗*^

Anxiety/depression (1–3)		2.48 ± 0.71	2.20 ± 0.81	*Z* = −2.11 (0.19/0.21); **p**=0.03^*∗*^	2.04 ± 0.63	1.35 ± 0.57	*Z* = −3.55 0.00 to 0.00; **p** < 0.01^*∗∗*^	*Z* = 2.31 0.01 to 0.01; **p** < 0.02^*∗*^	*Z* = −3.55 0.00 to 0.00; **p** < 0.01^*∗∗*^

VAS^†^ (0–100)		37.60 ± 18.77	55.60 ± 21.03	*Z* = −3.73 0.00 to 0.00; **p** < 0.01^*∗∗*^	49.57 ± 21.84	76.52 ± 15.84	*Z* = −4.03 0.00 to 0.00; **p** < 0.01^*∗∗*^	*Z* = −2.00 0.02 to 0.02; **p**=0.45^*∗*^	*Z* = −3.41 0.00 to 0.00; **p** < 0.01^*∗∗*^

Baropodometry— force plate (%)	0.06	65 ± 15.59	63.28 ± 14.20	*t* = −0.66 −3.59 to 7.03; *p*=0.51	69.83 ± 16.61	60.26 ± 13.97	*t* = 3.29 3.54 to 15.58; **p** < 0.01^*∗∗*^	*Z* = −1.13 0.12 to 0.13; *p*=0.25	*Z* = −1.43 0.07 to 0.08; *p*=0.15

Baropodometry—support surface (cm^2^)		105.24 ± 38.59	111.36 ± 45.43	*Z* = −0.91 0.18 to 0.19; *p*=0.36	105.96 ± 20.69	106.39 ± 27.37	*Z* = −0.15 0.42 to 0.44; *p*=0.87	*Z* = −0.76 0.22 to 0.23; *p*=0.44	*Z* = −.11 0.44 to 0.46; *p*=0.91

Note: ^*∗∗*^*p* < 0.01,  ^*∗*^*p* < 0.05. ^†^VAS: visual analog scale for health status; K-S: Kolmogorov–Smirnov; SD: standard deviation. CI 95%: 95% confidence interval. *N* = 48.

**Table 4:** Descriptive statistics (mean and standard deviation) of the intragroup variables in the experimental group: scale of satisfaction, adherence, and motivation in relation to training with video-game based therapy.

Dimensions (*n* = 23)	K-S†	Pre (mean ± SD)	Post (mean ± SD)	*p*
Motivation	0.20	17.70 ± 4.37	22.96 ± 2.53	*t* = −7.53; **p** < 0.01^*∗∗*^
Self-esteem	0.20	16.48 ± 3.26	21.91 ± 2.69	*t* = −9.61; **p** < 0.01^*∗∗*^
Adherence	0.20	19.22 ± 5.71	26.35 ± 3.29	*t* = −8.22; **p** < 0.01^*∗∗*^

^†^K-S: Kolmogorov–Smirnov. Note: ^*∗∗*^*p* < 0.01,  ^*∗*^*p* < 0.05.

## Data Availability

All data generated and analyzed during this study are included in this article.
